# Interaction between a diabetes-related methylation site (TXNIP cg19693031) and variant (GLUT1 rs841853) on fasting blood glucose levels among non-diabetics

**DOI:** 10.1186/s12967-022-03269-y

**Published:** 2022-02-14

**Authors:** Hao-Hung Tsai, Chao-Yu Shen, Chien-Chang Ho, Shu-Yi Hsu, Disline Manli Tantoh, Oswald Ndi Nfor, Shin-Lin Chiu, Ying-Hsiang Chou, Yung-Po Liaw

**Affiliations:** 1 Institute of Medicine, Chung Shan Medical University, Taichung City, 40201 Taiwan; 2 School of Medicine, Chung Shan Medical University, Taichung, 40201 Taiwan; 3 School of Medical Imaging and Radiological Sciences, Chung Shan Medical University, Taichung, 40201 Taiwan; 4 Department of Medical Imaging, Chung Shan Medical University Hospital, Taichung City, 40201 Taiwan; 5 Department of Physical Education, Fu Jen Catholic University, New Taipei, 24205 Taiwan; 6 Department of Public Health and Institute of Public Health, Chung Shan Medical University, No. 110 Sect. 1 Jianguo N. Road, Taichung, 40201 Taiwan; 7 Department of Ophthalmology, Changhua Christian Hospital, Changhua, Taiwan; 8 College of Nursing and Health Sciences, Da-Yeh University, Changhua, Taiwan; 9 Department of Radiation Oncology, Chung Shan Medical University Hospital, Taichung, 40201 Taiwan

**Keywords:** Fasting blood glucose, Non-diabetics, Thioredoxin-interacting protein, Glucose transporter 1, rs841853, cg19693031, Epigenetics, Genetic variants, Interaction

## Abstract

**Background:**

Type 2 diabetes mellitus (T2DM) is caused by a combination of environmental, genetic, and epigenetic factors including, fasting blood glucose (FBG), genetic variant rs841853, and cg19693031 methylation. We evaluated the interaction between rs841853 and cg19693031 on the FBG levels of non-diabetic Taiwanese adults.

**Methods:**

We used Taiwan Biobank (TWB) data collected between 2008 and 2016. The TWB data source contains information on basic demographics, personal lifestyles, medical history, methylation, and genotype. The study participants included 1300 people with DNA methylation data. The association of cg19693031 methylation (stratified into quartiles) with rs841853 and FBG was determined using multiple linear regression analysis. The beta-coefficients (β) and p-values were estimated.

**Results:**

The mean ± standard deviation (SD) of FBG in rs841853-CC individuals (92.07 ± 7.78) did not differ significantly from that in the CA + AA individuals (91.62 ± 7.14). However, the cg19693031 methylation levels were significantly different in the two groups (0.7716 ± 0.05 in CC individuals and 0.7631 ± 0.05 in CA + AA individuals (p = 0.002). The cg19693031 methylation levels according to quartiles were β < 0.738592 (< Q1), 0.738592 ≤ 0.769992 (Q1–Q2), 0.769992 ≤ 0.800918 (Q2–Q3), and β ≥ 0.800918 (≥ Q3). FBG increased with decreasing cg19693031 methylation levels in a dose–response manner (p_trend_ = 0.005). The β-coefficient was − 0.0236 (p = 0.965) for Q2–Q3, 1.0317 (p = 0.058) for Q1–Q2, and 1.3336 (p = 0.019 for < Q1 compared to the reference quartile (≥ Q3). The genetic variant rs841853 was not significantly associated with FBG. However, its interaction with cg19693031 methylation was significant (p-value = 0.036). Based on stratification by rs841853 genotypes, only the CC group retained the inverse and dose–response association between FBG and cg19693031 methylation. The β (p-value) was 0.8082 (0.255) for Q2–Q3, 1.6930 (0.022) for Q1–Q2, and 2.2190 (0.004) for < Q1 compared to the reference quartile (≥ Q3). The p_trend_ was 0.002.

**Conclusion:**

Summarily, methylation at cg19693031 was inversely associated with fasting blood glucose in a dose-dependent manner. The inverse association was more prominent in rs841853-CC individuals, suggesting that rs841853 could modulate the association between cg19693031 methylation and FBG. Our results suggest that genetic variants may be involved in epigenetic mechanisms associated with FBG, a hallmark of diabetes. Therefore, integrating genetic and epigenetic data may provide more insight into the early-onset of diabetes.

**Supplementary Information:**

The online version contains supplementary material available at 10.1186/s12967-022-03269-y.

## Background

Type 2 diabetes mellitus is a serious global public concern with a huge, yet constantly increasing burden. It negatively affects quality of life and greatly increases health care expenditure [1]. Type 2 diabetes affected approximately 462 million people (6.28% of the world’s population) in 2017 [2]. The disease is projected to affect 552 million people by 2030 [3]. The global prevalence rate was approximately 6059 patients per 100,000 in 2017 and is projected to be 7079 and 7862 cases per 100,000 by 2030 and 2040, respectively [2]. It accounted for more than 1 million deaths in 2017, making it the 9th cause of death in the world [2]. The continuous increase in the incidence, prevalence, and mortality of diabetes warrants urgent measures to prevent its occurrence in non-diabetics.

Type 2 diabetes has a multifaceted onset, encompassing a complex interplay of environmental, genetic, and epigenetic processes with poorly explained mechanisms [4–8]. The risk of diabetes is increased by hyperglycemia, increased age, high body mass index (BMI), dyslipidemia, unhealthy diet, and other lifestyle factors such as alcohol consumption and smoking [8–12]. Fasting blood glucose is an important indicator of diabetes, as well as a hallmark of diabetes management [6, 9, 12–14]. It is also a predictor of cardiovascular disease risk among diabetics and non-diabetics [15].

Glucose transporter 1 (or GLUT1), also called the solute carrier family 2, facilitated glucose transporter member 1 (SLC2A1) and thioredoxin-interacting protein (TXNIP) located on chromosome 1 are notable candidate genes for T2D diabetes [16–24]. GLUT1 is a well-characterized solute transporter that mainly regulates the cellular uptake of glucose in humans [13, 16]. TXNIP is an important modulator of glucose metabolism and mitochondrial activities associated with changing glucose levels [17–20]. TXNIP controls glucose uptake in cells, partly by binding to GLUT1, serving as a glucose-sensitive switch [17–20]. Its expression is a key element in glucose uptake mediated by GLUT1 [19].

Association studies and meta-analyses identified GLUT1 rs841853 single nucleotide polymorphism (SNP), also known as GLUT1 XbaI polymorphism as one of the genetic variants associated with diabetes and diabetic nephropathy, a diabetes-related complication [24–35].

DNA methylation, the most studied gene regulatory epigenetic process, is affected by environmental [6, 36, 37] and genetic factors [10, 38–40]. Perturbed DNA methylation influences gene expression [41, 42]. Several epigenome-wide association studies (EWAS) on methylation have identified TXNIP cg19693031 as the top diabetes-related methylation site [4, 22, 23, 43–48]. This site has also been associated with fasting blood glucose [22, 49].

DNA methylation is a strong disease marker that appears early during disease onset, especially cancer [50–52]. Genetic variants are population- and disease-specific. Hence, the identification of variants and biomarkers specific to certain diseases could be helpful in targeted therapy [53]. The genome intertwines with the epigenome [54] and there is a high probability that genomic variations cause diseases by affecting DNA methylation [55]. Therefore, the integration of genetic and methylation data could expand our understanding of disease etiology and prognosis. However, this area of research is lagging [54]. To our knowledge, no study has investigated the joint effect of genetic and epigenetic factors on diabetes and or FBG. In this regard and considering the important individual roles of TXNIP and GLUT1 in diabetes susceptibility and the direct interplay of both genes in glucose metabolism [17–20], we evaluated the interaction between the genetic variant (GLUT1 rs841853) and the epigenetic aberration (TXNIP cg19693031 methylation) on the fasting blood glucose levels of Taiwanese without type 2 diabetes. We hypothesized that the association between FBG and TXNIP cg19693031 methylation among non-diabetics may differ based on GLUT1 rs841853 genotypes.

## Materials and methods

### Study participants

We used TWB data collected between 2008 and 2016 for this study. The biobank contains data on basic demographics, personal lifestyle activities, dietary status, personal and family medical history, physical examination, methylation, and genotype. Taiwanese men and women aged 30 to 70 who have never had cancer were eligible to participate in the TWB project as explained previously [56, 57]. More information on the TWB can be found at https://www.twbiobank.org.tw/new_web_en/about-export.php. Data collection in the TWB was done following standard procedures. Initially, we recruited 1442 participants with data on methylation. However, we excluded people with diabetes (n = 115) and those with incomplete genotypes (n = 27). The final study participants included 1300 individuals. All participants provided written informed consents. This study was carried out in accordance with the Declaration of Helsinki and was approved by the Institutional Review Board of Chung Shan Medical University Hospital (CS1-2212).

### Definition of variables

In our study, diabetes was defined by (1) fasting blood glucose levels above 126 mg/dl, (2) hemoglobin A1c (HbA1c) levels of at least 6.5% [58], and (3) self-report of a previous clinical diagnosis with diabetes. We defined hypertension using the following criteria: (1) systolic blood pressure 140 mmHg; (2) diastolic blood pressure 90 mmHg) [59] or (3) self-report of prior hypertension diagnosis. The Hitachi LST008 analyzer was used to determine fasting blood glucose, high-density lipoprotein cholesterol (HDL-C), low-density lipoprotein cholesterol (LDL-C), and triglycerides. BMI was calculated using the standard formula: weight in kilograms divided by height in meters squared (kg/m^2^). Other variables were self-reported and were defined as follows: (1) cigarette smoking: habitual smoking for over 6 months; (2) alcohol drinking: habitual alcohol consumption of over 150 ml per week for the past 6 months; (3) regular exercise: any physical activity lasting over 30 min a day and taken at least three times a week; (4) tea drinking: habitual daily consumption of tea; (5) coffee drinking: consumption of coffee at least three times a week; (6) vegetarian: maintained a vegetarian diet for the past 6 months.

### SNP and DNA methylation assessments

We chose GLUT1 rs841853 and TXNIP cg19693031 due to their well-established links with T2DM. DNA methylation and SNP genotyping were conducted at Academia Sinica. Genotyping was done using custom TWB chips (versions 1 and 2) and imputed using SHAPEIT2 (v2.r790) and IMPUTE2 (v2.3.1) software. SNP genotyping and imputation details have been described elsewhere [60]. Briefly, the TWB version 1 chip contains about 650,000 SNPs for GWAS typed on the Axiom genome-wide CHB 1 Array plate (Affymetrix, Inc., Santa Clara, CA, USA). The TWB version 2 chip (Thermo Fisher Scientific, Inc., Santa Clara, CA, USA) can detect approximately 714,431 SNPs. GLUT1 rs841853 passed quality control tests: call rate > 95%, the Hardy–Weinberg equilibrium (p-value > 1.0 × 10^–6^), and a minor allele frequency > 0.01. DNA methylation was assessed with the Infinium MethylationEPIC BeadChipEPIC array (Illumina Inc. San Diego, CA, USA) using whole blood DNA treated with sodium bisulfite. The R software with the ChAMP package was used to process methylation data, including normalization and correction for batch effect and cell-type heterogeneity.

### Statistical analyses

We divided the TXNIP cg19693031 methylation levels into quartiles. The cut-offs were 0.738592 (25th percentile or Q1), 0.769992 (50th percentile or Q2), and 0.800918 (75th percentile or Q3). For GLUT1 rs841853, we used the dominant model and classified the genotypes into CC and CA + AA. Differences between discrete and continuous variables stratified by the GLUT1 rs841853 genotypes (CC and CA + AA) were compared using the Chi-square test and the t-test, respectively. The association of cg19693031 methylation, rs841853, and other variables with fasting blood glucose, as well as the interaction between cg19693031 methylation and rs841853 was determined by multiple linear regression analysis. We used ≥ Q3 as the reference group. Plink 1.9 and SAS 9.4 (SAS Institute, Cary, NC, USA) were used for data analyses.

## Results

Table 1 shows a summary of the demographic characteristics of the study participants classified into the rs841853 CC and CA + AA genotypes. There were 735 and 565 participants with CC and CA + AA genotypes, respectively. FBG levels (mean ± SD) between the genotypes (92.07 ± 7.78 for CC and 91.62 ± 7.14 for CA + AA) did not differ significantly. However, the cg19693031 methylation levels were significantly different in the two groups (0.7716 ± 0.05 in CC individuals and 0.7631 ± 0.05 in CA + AA individuals (p = 0.002). The cg19693031 methylation levels according to quartiles were β < 0.738592 (< Q1), 0.738592 ≤ 0.769992 (Q1–Q2), 0.769992 ≤ 0.800918 (Q2–Q3), and β ≥ 0.800918 (≥ Q3). Demographic characteristics of the study participants grouped into GLUT1 rs841853 genotype (CC, CA, and AA) are shown in Additional file 1: Table S1.

**Table 1:** Demographic characteristics of the study participants based on GLUT1 rs841853 genotypes (CC and CA + AA)

	rs841853-CC	rs841853-CA + AA	p-value
	(n = 735)	(n = 565)	
Fasting blood glucose (mg/dL)	92.07 ± 7.78	91.62 ± 7.14	0.278
TXNIP cg19693031 (β)	0.7716 ± 0.05	0.7631 ± 0.05	0.002
TXNIP cg19693031 quartiles			0.005
Q3 (β ≥ 0.800918)	210 (28.57)	117 (20.71)	
Q2–Q3 (0.769992 ≤ 0.800918)	187 (25.44)	140 (24.78)	
Q1–Q2 (0.738592 ≤ 0.769992)	168 (22.86)	158 (27.96)	
Q1 (β < 0.738592)	170 (23.13)	150 (26.55)	
Sex			0.309
Women	385 (52.38)	312 (55.22)	
Men	350 (47.62)	253 (44.78)	
Age (years)	48.87 ± 11.03	48.22 ± 11.06	0.288
BMI (kg/m^2^)	23.95 ± 3.36	24.31 ± 3.75	0.071
Cigarette smoking			0.191
No	559 (76.05)	447 (79.12)	
Yes	176 (23.95)	118 (20.88)	
Alcohol drinking			0.889
No	667 (90.75)	514 (90.97)	
Yes	68 (9.25)	51 (9.03)	
Triglyceride (mg/dL)	112.56 ± 95.94	112.60 ± 103.11	0.994
HDL-C (mg/dL)	55.96 ± 14.27	54.42 ± 13.70	0.051
LDL-C (mg/dL)	122.67 ± 32.36	123.38 ± 33.55	0.699
Hypertension			0.294
No	603 (82.04)	476 (84.25)	
Yes	132 (17.96)	89 (15.75)	
Regular exercise			0.709
No	420 (57.14)	317 (56.11)	
Yes	315 (42.86)	248 (43.89)	
Tea intake			0.460
No	451 (61.36)	358 (63.36)	
Yes	284 (38.64)	207 (36.64)	
Coffee intake			0.997
No	471 (64.08)	362 (64.07)	
Yes	264 (35.92)	203 (35.93)	
Vegetarian diet			0.405
No	671 (91.29)	588 (92.57)	
Yes	64 (8.71)	49 (7.43)	

Minimum and maximum TXNIP cg19693031 β = 0.510186 and 0.918557, respectively

Continuous variables are represented by mean ± SD and categorical variables by n (%)

*GLUT1* glucose transporter 1, *SD* standard deviation, *β* beta-value, *TXNIP* thioredoxin-interacting protein, *BMI* body mass index, *HDL-C* high-density lipoprotein cholesterol, *LDL-C* low-density lipoprotein cholesterol

Table 2 shows the association of cg19693031 methylation and the rs841853 variant with FBG. FBG increased with decreasing cg19693031 methylation levels in a dose–response manner (p_trend_ = 0.005). The β (p-value) was − 0.0236 (0.965) for Q2–Q3, 1.0317 (0.058) for Q1–Q2, and 1.3336 (0.019) for < Q1 compared to the reference quartile (≥ Q3). The rs841853 variant was not significantly associated with fasting blood glucose (β = − 0.4576, p = 0.232). However, its interaction with cg19693031 methylation (i.e., rs841853 genotypes*cg19693031 quartiles) was significant (p = 0.036) as shown in Table 3. Additionally, the additive model revealed a significant association between the AA genotype (reference: CC) and FBG (β = − 1.7643, p-value = 0.036) as shown in Additional file 1: Table S2.

**Table 2:** Association of TXNIP cg19693031 methylation and GLUT1 rs841853 with fasting blood glucose

	β	p-value
TXNIP cg19693031 (ref: ≥ Q3)		
Q2–Q3	− 0.0236	0.965
Q1–Q2	1.0317	0.058
< Q1	1.3336	0.019
*P for trend*		0.005
GLUT1 rs841853 (ref: CC)		
CA + AA	− 0.4576	0.232
Sex (ref: Women)		
Men	2.4784	< .001
Age	0.1935	< .001
BMI	0.1647	0.006
Cigarette smoking (ref: No)		
Yes	0.7118	0.166
Alcohol drinking (ref: No)		
Yes	1.2013	0.086
Triglyceride	0.0031	0.146
HDL-C	− 0.0338	0.037
LDL-C	0.0094	0.112
Hypertension (ref: No)		
Yes	0.3245	0.540
Exercise (ref: No)		
Yes	− 0.3790	0.362
Tea intake (ref: No)		
Yes	0.1308	0.742
Coffee intake (ref: No)		
Yes	− 0.2297	0.564
Vegetarian diet (ref: No)		
Yes	− 1.9887	0.004
TXNIP cg19693031*SLC2A1 rs841853	p-value = 0.036	

*TXNIP* thioredoxin-interacting protein, *GLUT1* glucose transporter 1, *β* beta coefficient, *ref* reference, *BMI* body mass index, *HDL-C* high-density lipoprotein cholesterol, *LDL-C* low-density lipoprotein cholesterol, *: interaction term

**Table 3:** Association between TXNIP cg19693031 methylation and fasting blood glucose stratified by GLUT1 rs841853 genotypes

	rs841853-CC		rs841853-CA + AA	
	β	p-value	β	p-value
TXNIP cg19693031 (ref: ≥ Q3)				
Q2–Q3	0.8082	0.255	− 1.1672	0.160
Q1–Q2	1.6930	0.022	0.0322	0.969
< Q1	2.2190	0.004	0.1037	0.905
*P for trend*		0.002		0.491
Sex (ref: Women)				
Men	2.5831	< .001	2.2765	0.001
Age	0.1863	< .001	0.1980	< .001

*TXNIP* thioredoxin-interacting protein, *GLUT1* glucose transporter 1, *β* beta coefficient, *ref* reference

Adjusted for body mass index, cigarette smoking, alcohol drinking, triglyceride, high-density lipoprotein cholesterol, low-density lipoprotein cholesterol, hypertension, regular exercise, vegetarian diet, and coffee and tea intake

When we examined the association between cg19693031 and FBG based on rs841853 genotypes (Table 3), the cg19693031 methylation levels were inversely associated with FBG in both groups but only the CC genotype showed significant results (β; p-value = 0.8082; 0.255 for Q2–Q3, 1.6930; 0.022 for Q1–Q2, and 2.2190; 0.004 for < Q1) compared to the reference quartile (≥ Q3). The trend test was significant only for the CC genotype (p_trend_ = 0.002).

When we combined the rs841853 genotypes and cg19693031 methylation quartiles using CC and ≥ Q3 as the reference group (Table 4), FBG levels were significantly higher among individuals carrying the CC genotype in the Q1–Q2 (β = 1.7709, p-value = 0.013) and < Q1 (β = 2.3116, p-value = 0.001) quartiles.

**Table 4:** Fasting blood glucose levels based on a combination of GLUT1 rs841853 genotypes and TXNIP cg19693031methylation quartiles

	β	p-value
GLUT1 rs841853 genotypes and TXNIP cg19693031 quartiles (ref: CC, ≥ Q3)		
CC, Q2–Q3	0.7815	0.254
CC, Q1–Q2	1.7709	0.013
CC, < Q1	2.3116	0.001
CA + AA, > Q3	1.2367	0.117
CA + AA, Q2–Q3	− 0.1411	0.849
CA + AA, Q1–Q2	1.0363	0.152
CA + AA, < Q1	1.0330	0.167

*GLUT1* glucose transporter 1, *TXNIP* thioredoxin-interacting protein, β: beta coefficient (denotes level of fasting blood sugar [mg/dL] for each category), ref: reference

Adjusted for sex, age, body mass index, cigarette smoking, alcohol drinking, triglyceride, high-density lipoprotein cholesterol, low-density lipoprotein cholesterol, hypertension, regular exercise, tea intake, coffee intake, and vegetarian diet

## Discussion

The heritability of diabetes is estimated at 20–80% [61, 62]. However, only 5–15% of this heritability has been explained [63]. Some methylation sites are believed to be heritable [64, 65]. Therefore, SNPs alone cannot fully delineate genetic heritability [6]. To our knowledge, this is the first study on blood sugar levels based on a genetic variant (rs841853) and an epigenetic modification (cg19693031 methylation) among non-diabetics.

In our study, we found significant differences in baseline TXNIP cg19693031 methylation levels between GLUT1 rs841853 genotypes (CC and CA + AA). There were no differences in FBG between these genotypes. Multiple linear regression analyses showed an inverse association between FBG and cg19693031 methylation levels in a dose–response manner. These results are consistent with those from numerous studies in which diabetes patients (higher FBG) had lower levels of TXNIP methylation [4, 22, 23, 43–49].

FBG did not differ between the GLUT1 rs841853 genotypes as previously reported [24]. The dominant model showed no significant association between rs841853-CA + AA genotype and FBG in our study. However, the additive model revealed a significant association between the rs841853-AA genotype and FBG. Previous literature contains contradictory findings regarding rs841853 and diabetes. For instance, rs841853 was not significantly associated with diabetes in several studies [24, 66–68]. Contrarily, the variant was confirmed as a diabetes-related SNP in Japanese [33, 69], Tunisian [34], and Bangladeshi [35] women. Some meta-analyses reported significant associations between rs841853 and diabetes [26, 30]. However, a meta-analysis found an association only among Asians, not Blacks or Caucasians. This suggests that the effect of the variant on T2DM varies across races [25]. Additional investigations of other diabetes-associated SNPs, including the recently reported polymorphism rs1800977 (C69T) within the ATP-binding cassette transporter A1 (ABCA1) [70] gene are necessary.

Even though the dominant model suggested that the rs841853-CA + AA genotype might not be independently associated with FBG in our study, the additive model showed a significant association between the AA genotype and FBG. Furthermore, the interaction between rs841853 and cg19693031 methylation was significant. The presence of a significant interaction between the genetic variant and the epigenetic process implies that rs841853 might be involved in the epigenetic mechanism (cg19693031 methylation) underlying diabetes. When we stratified the participants by the rs841853 genotypes, the dose–response and inverse association between FBG and cg19693031 methylation was retained only in the CC genotype. That is, in the presence of the CC genotype, lower levels of methylation (i.e., β < 0.738592 and 0.738592 ≤ 0.769992 corresponding to Q1–Q2 and < Q1) were associated with an increase (1.6930 for Q1–Q2 and 2.2190 for < Q1) in FBG levels. The increase in FBG (a hallmark of diabetes) indicates a higher probability of exposure to diabetes among non-diabetics with the rs841853 CC genotype who had lower levels of cg19693031 methylation. This could also imply that the GLUT1 rs841853 CC genotype and cg19693031 methylation might jointly influence the expression of TXNIP. However, we cannot clearly state the underlying mechanism. Notwithstanding, TXNIP is a gatekeeper for glucose metabolism which enhances glucose toxicity and pancreatic β cell apoptosis when highly expressed [17, 18, 20, 71, 72]. This gene is highly expressed in diabetes, which is characterized by impaired glucose-induced insulin production [71] and its inhibition could reduce glucotoxicity-related β-cell loss [73]. It is regarded as the main regulatory channel and an endocytosis adaptor for GLUT1 in glucose metabolism and the resulting mitochondrial actions in response to fluctuating glucose levels. That is, TXNIP is a signal regulation channel in glucose metabolism where it reduces glucose uptake by promoting GLUT1 endocytosis [18, 20].

An increase in GLUT1 mRNA expression is associated with an increase in glucose uptake [18]. However, TXNIP expression appears to be negatively associated with glucose levels and GLUT1. In the brain, GLUT1 is overly expressed, while TXNIP expression is very low [20]. TXNIP degradation resulting from glucose uptake was associated with the release of GLUT1 from endocytosis [17]. Furthermore, its overexpression in cultured adipocytes was associated with inhibited glucose uptake and vice versa [18]. TXNIP inhibits glucose influx directly or indirectly. The indirect mechanism involves the promotion of GLUT1 endocytosis by TXNIP that is transcriptionally induced by glucose [17, 18, 20]. The direct mechanism involves the binding of TXNIP to GLUT1 which inhibits the transport of glucose by GLUT1 at the plasma membrane [17]. In diabetes pathogenesis, slightly elevated blood sugar levels early in the disease onset enhance TXNIP expression and suppress glucose uptake by cells. This leads to increased blood sugar levels and subsequent overexpression of TXNIP, which down-regulates GLUT1 function and reduces glucose uptake in the periphery [18].

Our study was limited to participants without diabetes. However, when we included an additional model to determine FBG levels in diabetic patients (n = 114) based on GLUT1 TXNIP cg19693031 and variant rs841853 (data not shown), we found that (1) FBG levels decreased significantly with increasing methylation levels (β = − 377.4484, p < 0.001); (2) Compared to CC homozygotes, FBG levels were higher in patients with CA genotype (β = 11.0338) but lower in those with AA genotype (β = − 25.9662) even though these results were not significant (p > 0.05)*.* Despite these, selection bias cannot be ruled out due to the retrospective nature of our study.

DNA methylation is a strong disease marker that appears early during disease onset, especially cancer [50–52]. Genetic variants are population and disease-specific. Therefore, identifying specific variants and biomarkers for certain diseases could be useful in targeted therapy [53]. Therefore, monitoring the methylation patterns of diabetes-related genes in non-diabetics with a specific genetic variation could help in the identification of individuals at risk of diabetes.

## Conclusion

TXNIP cg19693031 methylation was inversely associated with FBG in a dose-dependent manner. The rs841853 variant was not independently associated with fasting blood glucose but had a significant interaction with cg19693031 methylation. The inverse association between TXNIP cg19693031 methylation and FBG became more prominent in the presence of the GLUT1 rs841853 CC genotype, suggesting that rs841853 could modulate the association between cg19693031 methylation and FBG. Both genetic variants and epigenetic changes associated with FBG could help in the early identification of individuals at risk of T2DM. Hence, considering the methylation patterns of diabetes-related genes in non-diabetics with specific genetic variants is essential when investigating the pathogenesis of T2DM.

## Supplementary Information

**Additional file 1: Table S1. **Demographic characteristics of the study participants grouped by GLUT1 rs841853 genotypes (CC, CA, and AA). **Table S2.** Association of TXNIP cg19693031 methylation and GLUT1 rs841853 genotype with fasting blood glucose (additive model).

## Data Availability

The data that support the findings of this study are available from Taiwan Biobank but restrictions apply to the availability of these data, which were used under license for the current study, and so are not publicly available. Data are, however, available from the authors upon reasonable request and with permission of Taiwan Biobank.
