# Supplementary material for: Interaction between a diabetes-related methylation site (TXNIP cg19693031) and variant (GLUT1 rs841853) on fasting blood glucose levels among non-diabetics
Source: J Transl Med. 2022 Feb 14;20:87. doi: 10.1186/s12967-022-03269-y (PMC8842527; doi:10.1186/s12967-022-03269-y)
Supplement: Supplementary file 1 — Additional file 1: Table S1. Demographic characteristics of the study participants grouped by GLUT1 rs841853 genotypes (CC, CA, and AA). Table S2. Association of TXNIP cg19693031 methylation and GLUT1 rs841853 genotype with fasting blood glucose (additive model). [file 12967_2022_3269_MOESM1_ESM.docx]

Table S1. Demographic characteristics of the study participants grouped by GLUT1 rs841853 genotypes (CC, CA, and AA)

|  | CC | |  | CA | |  | AA | | p-value |
| --- | --- | --- | --- | --- | --- | --- | --- | --- | --- |
|  | (n = 735) | |  | (n = 493) | |  | (n = 72) | |  |
| Fasting glucose (mg/dL) | 92.07 | ±7.78 |  | 91.82 | ±7.08 |  | 90.28 | ±7.47 | 0.151 |
| TXNIP cg19693031 (β) | 0.7716 | ±0.05 |  | 0.7616 | ±0.05 |  | 0.7734 | ±0.05 | 0.001 |
| TXNIP cg19693031 quartiles |  |  |  |  |  |  |  |  |  |
| Q3 (β ≥0.800918) | 210 | (28.57) |  | 100 | (20.28) |  | 17 | (23.61) | 0.025 |
| Q2-Q3 (0.769992≤0.800918) | 187 | (25.44) |  | 120 | (24.34) |  | 20 | (27.78) |  |
| Q1-Q2 (0.738592≤0.769992) | 168 | (22.86) |  | 138 | (27.99) |  | 20 | (27.78) |  |
| Q1(β <0.738592) | 170 | (23.13) |  | 135 | (27.38) |  | 15 | (20.83) |  |
| Sex |  |  |  |  |  |  |  |  | 0.595 |
| Women | 385 | (52.38) |  | 272 | (55.17) |  | 40 | (55.56) |  |
| Men | 350 | (47.62) |  | 221 | (44.83) |  | 32 | (44.44) |  |
| Age (years) | 48.87 | ±11.03 |  | 48.31 | ±11.10 |  | 47.57 | ±10.85 | 0.493 |
| BMI (kg/m^2^) | 23.95 | ±3.36 |  | 24.22 | ±3.70 |  | 24.92 | ±4.06 | 0.055 |
| Cigarette smoking |  |  |  |  |  |  |  |  | 0.352 |
| No | 559 | (76.05) |  | 388 | (78.70) |  | 59 | (81.94) |  |
| Yes | 176 | (23.95) |  | 105 | (21.30) |  | 13 | (18.06) |  |
| Alcohol drinking |  |  |  |  |  |  |  |  | 0.967 |
| No | 667 | (90.75) |  | 448 | (90.87) |  | 66 | (91.67) |  |
| Yes | 68 | (9.25) |  | 45 | (9.13) |  | 6 | (8.33) |  |
| Triglyceride (mg/dL) | 112.56 | ±95.94 |  | 112.97 | ±107.70 |  | 110.06 | ±63.68 | 0.973 |
| HDL-C (mg/dL) | 55.96 | ±14.27 |  | 54.71 | ±13.79 |  | 52.46 | ±12.95 | 0.066 |
| LDL-C (mg/dL) | 122.67 | ±32.36 |  | 122.80 | ±33.40 |  | 127.36 | ±34.60 | 0.507 |
| Hypertension |  |  |  |  |  |  |  |  | 0.572 |
| No | 603 | (82.04) |  | 415 | (84.18) |  | 61 | (84.72) |  |
| Yes | 132 | (17.96) |  | 78 | (15.82) |  | 11 | (15.28) |  |
| Regular exercise |  |  |  |  |  |  |  |  | 0.143 |
| No | 420 | (57.14) |  | 269 | (54.56) |  | 48 | (66.67) |  |
| Yes | 315 | (42.86) |  | 224 | (45.44) |  | 24 | (33.33) |  |
| Tea intake |  |  |  |  |  |  |  |  | 0.603 |
| No | 451 | (61.36) |  | 315 | (63.89) |  | 43 | (59.72) |  |
| Yes | 284 | (38.64) |  | 178 | (36.11) |  | 29 | (40.28) |  |
| Coffee intake |  |  |  |  |  |  |  |  | 0.999 |
| No | 471 | (64.08) |  | 316 | (64.10) |  | 46 | (63.89) |  |
| Yes | 264 | (35.92) |  | 177 | (35.90) |  | 26 | (36.11) |  |
| Vegetarian diet |  |  |  |  |  |  |  |  | 0.530 |
| No | 671 | (91.29) |  | 458 | (92.90) |  | 65 | (90.28) |  |
| Yes | 64 | (8.71) |  | 35 | (7.10) |  | 7 | (9.72) |  |

Minimum and maximum TXNIP cg19693031 β = 0.510186 and 0.918557, respectively.

Continuous variables are represented by mean±SD and categorical variables by n(%).

GLUT1: glucose transporter 1, SD: standard deviation, β = beta-value, TXNIP: thioredoxin-interacting protein, BMI: body mass index, HDL-C: high-density lipoprotein cholesterol, LDL-C: low-density lipoprotein cholesterol.

| Table S2. Association of TXNIP cg19693031 methylation and GLUT1 rs841853 genotype with fasting blood glucose | | |
| --- | --- | --- |
|  | β | p-value |
| TXNIP cg19693031 (ref: ≥Q3) |  |  |
| Q2-Q3 | -0.0252 | 0.962 |
| Q1-Q2 | 1.0182 | 0.061 |
| <Q1 | 1.2996 | 0.022 |
| *P for trend* |  | 0.006 |
| SLC2A1 rs841853 (ref: CC) |  |  |
| CA | -0.2674 | 0.502 |
| AA | -1.7643 | 0.036 |
| Sex (ref: Women) |  |  |
| Men | 2.4830 | <.0001 |
| Age | 0.1936 | <.0001 |
| BMI | 0.1682 | 0.005 |
| Cigarette smoking (ref: No) |  |  |
| Yes | 0.6876 | 0.181 |
| Alcohol drinking (ref: No) |  |  |
| Yes | 1.1984 | 0.087 |
| Triglyceride | 0.0030 | 0.156 |
| HDL-C | -0.0346 | 0.033 |
| LDL-C | 0.0096 | 0.103 |
| Hypertension (ref: No) |  |  |
| Yes | 0.3223 | 0.542 |
| Exercise (ref: No) |  |  |
| Yes | -0.4133 | 0.320 |
| Tea intake (ref: No) |  |  |
| Yes | 0.1436 | 0.718 |
| Coffee intake (ref: No) |  |  |
| Yes | -0.2301 | 0.563 |
| Vegetarian diet (ref: No) |  |  |
| Yes | -1.9699 | 0.005 |
